# Effects of social support, hope and resilience on depressive symptoms within 18 months after diagnosis of prostate cancer

**DOI:** 10.1186/s12955-020-01660-1

**Published:** 2021-01-07

**Authors:** Xinxin Zhao, Ming Sun, Ye Yang

**Affiliations:** 1 Department of Hospice, Shengjing Hospital of China Medical University, Shenyang, 110004 China; 2 Department of Urology, Shengjing Hospital of China Medical University, Shenyang, China; 3 Department of Ultrasound, Shengjing Hospital of China Medical University, Shenyang, China

**Keywords:** Prostate cancer, Social support, Hope, Resilience, Depressive symptoms

## Abstract

**Background:**

The prevalence of depression symptoms and related modifiable factors in prostate cancer (PCa) are not well evaluated. We aimed to assess the effects of perceived social support, hope and resilience on depressive symptoms within 18 months after diagnosis of PCa, and to evaluate the role of hope and resilience as mediators of that relationship.

**Method:**

A cross-sectional study was analyzed in consecutive inpatients with PCa during the months of January 2018 and August 2019. A total of 667 patients eligible for this study completed questionnaires on demographic and clinic variables, Center for Epidemiologic Studies Depression Scale, Multidimensional Scale of Perceived Social Support, Adult Hope Scale, and Resilience Scale (14 items). All registered patients were all volunteers and anonymous. Depressive symptoms, perceived social support, hope and resilience were measured anonymously. Out of 667 patients, a total of 564 effective respondents (< 30% missing data) became our subjects. Hierarchical linear regression was used to identify the factors associated with depressive symptoms. Asymptotic and resampling strategies were used to conduct the mediating effects of hope and resilience.

**Results:**

The prevalence of depressive symptoms was 65.9% in PCa patients. Hierarchical regression analyses indicated that perceived social support, hope, and resilience together accounted for 27.5% variance of depressive symptoms. Support from family, hope, and resilience significantly associated with depressive symptoms, respectively. Hope (a*b = − 0.0783, BCa95% CI: − 0.134 to − 0.0319, *p* < 0.05), and resilience (a*b = − 0.1315, BCa95% CI: − 0.1894 to − 0.0783, *p* < 0.05) significantly mediated the association between perceived social support and depressive symptoms.

**Conclusions:**

The high prevalence of depressive symptoms among PCa patients should receive more attention. Perceived social support, hope and resilience could be positive resources for combating depressive symptoms, and hope and resilience mediated the association between perceived social support and depressive symptoms. Enhancing social support, particularly the support form family, and improving patients’ outlook and resilience may be potential targets for future psychosocial interventions aimed at reducing depressive symptoms.

## Introduction

Prostate cancer (PCa) is the second commonest diagnosed malignancy and the fifth leading cause of cancer mortality in men, accounting for a substantial public health burden [1]. Similar to other Asia countries where PCa incidence and mortality have been historically low, the trend of PCa incidence and mortality has experienced significant increases in China, and the average age at diagnosis declined slightly [2, 3]. Additionally, especially for the unique concerns in Chinese PCa patients, high medical costs and insufficient insurance coverage make the treatment of cancer a catastrophic event for Chinese families. Both the incidence and mortality of PCa also showed significant increase in China [4]. PCa symptoms and side effects of treatment include pain, fatigue, and impairment in urinary and sexual functioning [5]. Therefore, in addition to mortality concerns, men with PCa are at risk for psychological distress, and one aim of our studies was to address the specific psychological concerns associated with the diagnosis and treatment of PCa.

Previous studies have found an increased risk of depressive symptoms, hospitalization for depression, and use of antidepressant medication in PCa population [6, 7]. Concurrent with PCa, depression has been associated with a lower adherence to treatment, increased periods of hospitalization and decreased overall survival [8–10]. Watts et al. identified pretreatment, on-treatment and post-treatment depression rates of 17.27%, 14.70% and 18.44%, suggesting that prevalence of depression in men with PCa, across the treatment spectrum, is relatively high [6]. With the advances in treatment, there has been an increasing interest about psychological distress in PCa with androgen deprivation therapy [11] and survivors [12], but not much is generally known regarding the mental health issues of patients within 18 months after diagnosis of PCa.

In order to confront depression in PCa patients, internal adaptation for stress and external support from the society play important roles for individuals to overcome their depression [13, 14]. As Snyder et al. noted, hope is identified as a positive motivational state that consists of two parts: (1) Agency: perception about one’s ability to initiate and sustain motivation toward a goal; (2) Pathways: thinking about the methods or routs of reaching the desired goals [15]. Hope is an important and positive factor in their lives of cancer patients [16]. In light of the challenges PCa patients face, they who have a higher sense of determination to meet the goals that they set (agency) as well as successful planning of ways to meet their goals (pathways) might appear to be better equipped to adjust well to these stressors.

Resilience has been introduced in referring protective individual attributes in the adaptation to cancer [14, 17], which is defined as an individual’s capacity to maintain psychological/physical well-being in the face of adversities [18]. Increased resilience plays a vital role in eliminating the stress encountered in cancer [19, 20], and the internal mechanisms have been suggested (e.g., “bouncing back” from adversity and the use of adaptive coping strategies) [17, 18]. Resilience is associated with lower psychological distress, better adjustment, and better quality of life (QoL) among cancer patients [20–22]. Therefore, resilience might be essential for patients to relieve depression caused by PCa-related stressors.

A useful way to investigate the role that hope and resilience play in the face of cancer is to put them in a context of social support [21, 23, 24]. Social support could protect cancer patients from the adverse psychological effects, and act as buffer against cancer-related stress [21, 25, 26]. Among non-cancer populations, hope and resilience significantly mediated the relations between social support and depression [27, 28]. Several studies also found the mediating roles of coping strategies and perceived stress between social support and QoL in cancer patients [29, 30]. The above studies suggested that social support not only revealed a direct effect but also exerted an indirect effect on depression through triggering mediators. Additionally, hope and resilience have been proven to mediate the association between antecedent variables and emotional outcomes in cancer patients [31, 32]. Nevertheless, the roles of social support, hope and resilience in combating depression have not been studied among PCa patients. Besides, whether hope and resilience mediates the association between social support and depression has not been evaluated.

In light of the above concerns, the aim of the present study was to assess the depression among PCa patients as well as to explore the protective effects of social support, hope and resilience on depression within the first 18 months of diagnosis. More importantly, we aimed to confirm whether hope and resilience mediated the association between social support and depression. The first 18 months of diagnosis was chosen because most studies mainly focused on the 1, 6 and 12 months after surgery, we extended the investigation time appropriately to capture the emotional state of patients.

## Method

### Participants and procedures

A cross-sectional study was analyzed in consecutive inpatients with PCa during January 2018 and August 2019. The study took place at the Department of Urology in our Hospital, which is the main provider of cancer services to a geographically defined area of 8.2 million people. The eligibility criteria for patient recruitment were (1) age 18 years or older, (2) being histologically diagnosed with PCa, (3) aware of the cancer diagnosis, (4) able to understand and read Chinese well enough to answer the questionnaires, (5) time since diagnosis ≤ 18 months. Exclusion criteria were the following: (1) patients had a history of psychiatric problems before cancer diagnose, (2) patients had intellectual and/or cognitive impairments, (3) patients had other active cancers.

Consecutive patients from the Urology wards were potentially eligible, unless they demonstrated unwillingness to participate. The patients’ attending physicians discussed eligibility on a case-by-case basis to avoid biased judgment and selection bias (e.g., interacting with patients face to face based on the inclusion and exclusion criteria). All registered patients were all volunteers and anonymous for investigators. After obtaining written consent, patients were asked about socio-demographic characteristics, including ability to read and number of years’ formal schooling. Clinical data was collected from the medical record and a set of self-report questionnaires were distributed to patients at the time of hospitalization. Data was mainly obtained using self-administered questions, so there was a possibility of recall and reporting bias.

Among a total of 667 registered patients, 10 patients refused to participate, and 5 patients had other active cancers. Of 652 eligible patients for this study, 88 were excluded from analysis (> 30% missing data). Finally, we received effective responses from 564 PCa patients with effective response rate 86.5%. Medical Ethics Committee of Shengjing Hospital Affiliated to China Medical University reviewed this study, provided the ethics for the approval of this study, and determined that the study procedures were in accordance with the ethical standards.

## Questionnaires

### Demographic and cancer-related variables

The demographic variables included age, marital status and education. Time since diagnosis, cancer stage, treatment type and metastasis (yes vs. no) were included as the clinical variables. They were mainly collected by medical record and questionnaires.

### Depression

Depression was measured by the Center for Epidemiologic Studies Depression Scale (CES-D), which is a 20-item measure of the severity of depressive symptoms [33]. Items are ranked on a four-point frequency scale from 0 (never) to 3 (always). Higher scores reflect worse depressive symptoms, and a score ≥ 16 indicates probable clinical depression [33]. The Chinese version of CES-D was validated for criterion, content, reliability and convergent [34, 35]. The advantages of CES-D are that patients could complete these scales in a shorter period of time, which is very important given their diseases and physical/mental states. For the disadvantages, CES-D is a screening and non-diagnostic measure. It should be more cautious throughout the study to state the findings about the prevalence of depression. Cronbach’s alpha for CES-D was 0.794 in this study.

### Perceived social support

Perceived social support was measured by the Multidimensional Scale of Perceived Social Support (MSPSS) [36], which is 12-item measure of the sources of perceived social support, rated on a seven-point scale from 1 (very strongly disagree) to 7 (very strongly agree). It provides a summary score (12 to 84), as well as three subscales for perceived support from family, friends, and significant others. The MSPSS was validated and commonly used in Chinese cancer patients [20, 21, 26]. In this study, the Cronbach’s alpha was 0.942, 0.923, 0.896, and 0.931 respectively for MSPSS, family, friends, and significant others subscales.

### Hope

For hope, we correlated this with “hope” and this was measured by the Adult Hope Scale (AHS) which included eight items and four filler items rated on 4-point scales (1 = strongly disagree, 4 = strongly agree) [15]. The AHS contains four Agency and four Pathways items, and a high score denotes a higher level of pathways and agency. The hope level is the sum of the agency and pathways items. The AHS and its Chinese version have been used in cancer patients with acceptable validity and reliability [20, 21]. The Cronbach’s alpha was 0.742, 0.772, and 0.849 respectively for the pathway, agency and AHS.

### Resilience

The 14-items version of Resilience Scale (RS-14), a short version of the original RS (i.e. RS-25), was used to assess resilience [37]. RS-14 consists of 14 items rated on a 7-point scale, ranging from 1 (strongly disagree) to 7 (strongly agree). The total score ranges from 14 to 98 scores, with higher scores indicating higher resilience. The Chinese version of RS-14 had a good validity and reliability among cancer patients [20, 21, 38]. In this study, the Cronbach’s alpha was 0.959 for RS-14.

## Statistical methods

The Statistical Package for the Social Sciences (SPSS, version 13.0) was used to perform the statistical analyses, with two-tailed probability value of < 0.05 considered to be statistically significant. The distributions of CES-D in categorical variables were calculated using independent sample *t*-test and one way analysis of variance (ANOVA). When one-way ANOVA was found to be significant, least-significant-difference (LSD) was done to perform multiple comparisons. Pearson’s correlation was used to examine correlations among psychosocial variables. Hierarchical regression analysis was used to explore the effects of perceived social support, hope and resilience on depression with adjustment for demographics and clinical variables related to depression in univariate analysis (*p* < 0.05). There were two models (Model 1 and Model 2) in Step 2. Total score of MSPSS was added in Model 1, and three subscales of MSPSS (others, friend and family) were added in Model 2. Due to the high correlations among the MSPSS subscales, these variables were adjusted in the stepwise regression in Step 2 (Model 2).We provided data including R^2^, adjusted R^2^ (Adj.R^2^), R^2^-changes, F, standardized regression coefficient (β) and *p* value for each step in the regression model. Asymptotic and resampling strategies were used to examine the mediating roles (a*b product) of hope and resilience on the association between perceived social support and depression [39]. In these equations, perceived social support was modeled as the independent variable, CES-D score as the dependent variable, hope and resilience as the mediators. The auxiliary routine estimate was based on 5000 bootstrap samples. Then, the bias-corrected and accelerated 95% confidence interval (BCa95% CI) for each a*b product was investigated, and a BCa95% CI not including 0 indicated a significant mediating role. All study variables were centralized before analysis to account for differences in scale scores. Moreover, tolerance (> 0.10) and variance inflation factor (< 10) were used to check for multicollinearity.

## Results

In the present study, the patients (N = 564) were in the age range of 18–80 (Mean ± SD: 59.66 ± 11.21), and the number of months after diagnosis was in the range of 1–18 (Mean ± SD: 11.76 ± 22.93). Demographic and clinical factors of patients and distributions of depressive symptoms in categorical items were shown in Table 1. Patients with a higher level of education had a lower level of depressive symptoms. Results also indicated that patients whose time since diagnosis was within 3 months had a higher level of depressive symptoms as well as patients at cancer stage II had higher scores of CES-D.

**Table 1:** CES-D scores in demographic and clinical variables (N = 564)

	N (%)	CES-D	t/F value	*p*-value
*Demographic variables*				
Age			0.482	0.547
≤ 55	227 (40.2)	22.53 ± 9.54		
56–65	193 (34.2)	23.77 ± 8.34		
≥ 66	144 (25.6)	23.32 ± 9.86		
Marital status			0.574	0.532
Married/living with a partner	495 (87.8)	23.07 ± 9.16		
Single/widowed/divorced	69 (12.2)	23.89 ± 9.50		
Education			8.903	< 0.001
Middle school or below	265 (46.9)	24.29 ± 9.12^b^		
High school	144 (25.5)	24.37 ± 8.16^b^		
Junior college or above	156 (27.6)	20.48 ± 9.32^a^		
*Clinical variables*				
Time since diagnosis			3.021	0.025
≤ 3	143 (25.3)	25.09 ± 8.36^a^		
4–6	139 (24.6)	23.35 ± 9.02		
7–12	194 (34.4)	22.14 ± 10.09^b^		
13–18	88 (15.6)	22.51 ± 8.84		
Cancer stage			− 2.245	0.007
I	298 (52.8)	22.31 ± 9.41^a^		
II	266 (47.2)	24.47 ± 8.82^b^		
Treatment type			− 1.207	0.214
No treatment	5 (0.8)	–		
Surgery	290 (51.4)	22.97 ± 9.36		
Combined treatment^c^	269 (47.7)	23.54 ± 8.28		
Chemotherapy	2 (0.2)	–		

*CES-D* Center for Epidemiologic Studies Depression Scale

^a,b^Calculated by least-significant-difference (LSD), mean scores for depression with unequal superscripts differ significantly at the *p* < 0.05 level

^c^Combined treatment included surgery and other treatments (radiation and/or endocrinotherapy)

In Table 2, Pearson’s correlation coefficients were calculated among study variables. Perceived social support, hope and resilience was negatively associated with depressive symptoms (r = ranged from − 0.337 to − 0.447; *p* < 0.01). Additionally, the prevalence of depressive symptoms in PCa patients was 65.9% (N = 372).

**Table 2:** Means, standard deviation, range and zero-order correlations (Pearson’s r) among study variables

Variables	CES-D scores ≥ 16	Mean ± SD	Range	1	2	3	4	5	6	7
1. CES-D	372(65.9%)	23.72 ± 9.24	0–44	1	− 0.398**	− 0.364**	− 0.404**	− 0.337**	− 0.424**	− 0.447**
2. Total-MSPSS		58.07 ± 16.25	20–84		1	0.959**	0.961**	0.923**	0.467**	0.503**
3. MSPSS-others		19.58 ± 6.02	4–28			1	0.912**	0.834**	0.442**	0.484**
4. MSPSS-family		20.37 ± 6.62	6–28				1	0.834**	0.475**	0.493**
5. MSPSS-friend		19.05 ± 5.74	6–28					1	0.483**	0.465**
6. AHS		21.74 ± 4.21	8–32						1	0.546**
7.RS-14		65.58 ± 17.03	14–98							1

*SD* standard deviation, *CES-D* Center for Epidemiologic Studies Depression Scale, *MSPSS* Multidimensional Scale of Perceived Social Support, *AHS* Adult Hope Scale, *RS-14* 14-items version of Resilience Scale

^**^Correlation is significant at the 0.01 level (two-tailed)

Hierarchical regression analysis results were presented in Table 3. Psychosocial variables together accounted for an additional 27.5% variance to the prediction of depression. In Step 2 Model 1, perceived social support was significantly and negatively associated with depressive symptoms (β = − 0.377, *p* < 0.001). In Step 2 Model 2, perceived social support-family was significantly and negatively associated with depressive symptoms (β = − 0.387, *p* < 0.001). Hope and resilience were significantly and negatively associated with depressive symptoms in Step 3 Model 1 and Model 2. In addition, the effect of perceived social support on depressive symptoms in Step 3 was reduced compared with that in Step 2, as indicated by smaller β coefficients. Tolerance (range: 0.573–0.986) and variance inflation (range: 1.014–1.797) did not indicate an obvious multicollinearity problem.

**Table 3:** Results from the hierarchical regression analyses

Variables	Step1(β)	Step2(β)		Step3(β)	
		Model 1	Model 2	Model 1	Model 2
Covariates					
Age	0.035	0.031	0.039	0.034	0.037
Education1	0.196***	0.104*	0.097*	0.049	0.045
Education2	0.172**	0.103*	0.107*	0.044	0.046
Time since diagnosis	− 0.027	0.023	0.028	0.021	0.026
Cancer stage	0.106*	0.064	0.063	0.054	0.055
Social support					
Perceived social support		− 0.377***		− 0.168***	
Perceived social support-others			–		–
Perceived social support-family			− 0.387***		− 0.183***
Perceived social support-friend			–		–
Psychological resource					
Hope				− 0.176***	− 0.175***
Resilience				− 0.270***	− 0.264***
F	4.247***	17.064***	17.681***	24.648***	25.229***
R^2^	0.044	0.177	0.182	0.286	0.291
Adj.R^2^	0.033	0.166	0.172	0.275	0.279
R^2^-changes	0.044	0.133	0.138	0.109	0.109

β = standardized regression coefficient; Education1 = Middle school or below vs. Junior college or above; Education2 = High school vs. Junior college or above; Adj.R^2^ = adjusted R^2^

There were two models (Model 1 and Model 2) in Step 2. Perceived social support (total score) was added in Model 1, and its components were added in Model 2 adjusted by the stepwise regression due to the high correlations among the MSPSS subscales

*p < 0.05, ** *p* < 0.01, *** *p* < 0.001

**Fig. 1:**
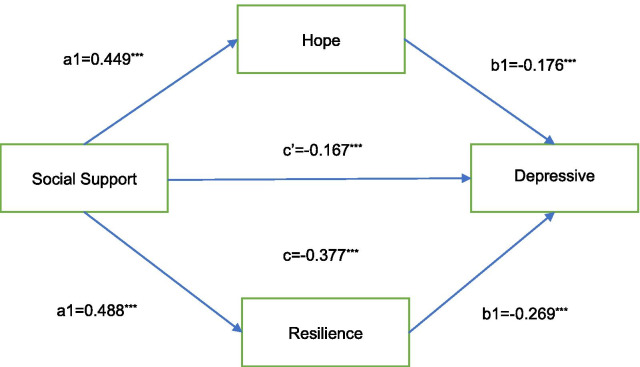
Indirect pathways of social support with depressive symptoms through hope and resilience **a** associations of social support with hope/resilience; **b** associations of hope/resilience with depressive symptoms after controlling for social support; **c** associations of social support with depressive symptoms; **c**′ associations of social support with depressive symptoms after adding hope/resilience as mediators. ***p < 0.001.

Path coefficients (a) (between social support and mediators) and (b) (between mediators and depressive symptoms), a*b products, and BCa 95% CI for these products are presented in Table 4 and Fig. 1. Perceived social support was significantly and positively associated with hope and resilience. Consistent with the results from hierarchical regression, hope and resilience were significantly and negatively associated with depressive symptoms after controlling for covariates. Thus, significant mediating roles of hope (a*b = − 0.0783, BCa95% CI: − 0.134 to − 0.0319, p < 0.05) and resilience (a*b = − 0.1315, BCa95% CI: − 0.1894 to − 0.0783, p < 0.05) on the association between perceived social support and depressive symptoms were revealed. The same conclusion also applies to perceived support from family considered as independent variable.

**Table 4:** Multiple mediation of the indirect effects of perceived social support on depressive symptoms through changes in hope and resilience (n = 564; 5000 bootstrap resamples)

Independent variable X	Mediators M	Dependent variable Y	Effect of X on M(a)	Effect of M on Y(b)	Indirect effect (a*b)^b^	BCa95% CI^a^		Total indirect effect (a*b)^b^	BCa95% CI^a^		Direct effect (c’**)**	Total effect (c)
						Low	High		Low	High		
Social support	Hope resilience	Depression	0.4490***	− 0.1764***	− 0.0783	− 0.1341	− 0.0319	− 0.2098	− 0.2734	− 0.1524	− 0.1668***	− 0.3776***
			0.4878***	− 0.2698***	− 0.1315	− 0.1894	− 0.0783					
Social support-family	Hope resilience	Depression	0.4311***	− 0.1753***	− 0.0754	− 0.1256	− 0.0320	− 0.2014	− 0.2682	− 0.1434	− 0.1842***	− 0.3857***
			0.4762***	− 0.2645***	− 0.1261	− 0.1847	− 0.0764					

All the coefficients are standardized regression coefficients and adjusted for age, education, cancer stage and time since diagnosis

***p < 0.001

^a^BCa = Bias corrected and accelerated bootstrapping confidence intervals that include corrections for both median bias and skew. BCa95%Confidence intervals (BCa95%CI) containing 0 is interpreted as not significant

^b^Bootstrap results for indirect effects after bias corrected

## Discussion

Results indicated that PCa patients in China suffered from depressive symptoms, and perceived social support (especially support from family), hope and resilience can be positive resources for reducing depressive symptoms. This is the first study to verify the mediating roles of a hope and resilience on the association between perceived social support and depressive symptoms using a relatively large sample (N = 564).

The prevalence of depressive symptoms was 65.9% in our study. We compared our results with other studies of depression in PCa patients using the same cut-off: (1) Hoyt and Carpenter reported the prevalence of depressive symptoms (23%, N = 15) in patients with radical prostatectomy or radiation therapy [40]; (2) Lin et al. found that 75 Taiwan patients (56.4%) had depressive symptoms [41]; (3) The prevalence of depressive symptoms among Kinlock’s sample of Black men was approximately 33% [42]. Men transitioning from healthy person to PCa patients might face intense physiological, psychological and interpersonal challenges after the cancer diagnosis. These unique characteristics, combined with the psychological issues that received little attention in mainland Chinese cancer patients [43], might aggravate PCa patients’ psychological problem.

Our results indicated that an increase in perceived social support has some correlation with depressive symptoms yet hope and resilience were negatively associated with depressive symptoms. Social support decreased cancer-related stress and had the potential for combating psychological problems [21, 25, 26, 30], and our results were consistent with previous studies. However, only social support from family was significantly associated with depressive symptoms. This could be attributed to the fact that family is the bedrock of Chinese society, and the care and concern of family members are of great importance for cancer patients. Psychosocial interventions involving family members have been also proven to be beneficial for depression in cancer patients [44]. Additionally, due to the changed self-image/body image and altered sexual/urinary function, PCa patients might not ask for support from friends or significant others, and may distance themselves from friends and family members.

After controlling for social support, hope and resilience also accounted for an additional proportion of variance to depressive symptoms (10.9%). Regarded as positive psychological resources, hope and resilience have been proven to be beneficial in cancer patients. Hope might provide cancer patients positive coping strategies for depression, including sustaining the movement toward achieving a goal and providing the pathways of reaching the desired goals [13, 16]. Resilient patients might show more emotional stability when faced with adversity and obstacle caused by cancer [14, 17]. These findings prompted us to believe that both positive experiences regarding one’s own goal and route (hope) and positive adaptation in the context of traumatic events (resilience) were important to effectively ameliorate and even overcome depressive symptoms in PCa patients.

An important finding was that hope and resilience mediated the negative relationship between perceived social support and depressive symptoms using the non-parametric bootstrapping procedure. Besides the direct effect of perceived social support on depression, PCa patients who perceive more social support, especially support from family, might be more likely to experience higher hope and resilience, which in turn reduced their depressive symptoms. Additionally, the indirect effect of resilience was larger than that of hope in the multiple mediators analysis, indicating the importance of patients’ capacity to maintain and recover the psychological well-being in the face of cancer.

## Implications

The present study indicated, that more effort should be devoted to improve social support (especially family support), as well as to elevate hope and resilience in PCa patients. Provision of social support to family of PCa patients could be substantial in reducing depressive symptoms. Family members also should not give up providing reassurance and spending time with patients [45]. In addition, Berendes et al. developed a psychological intervention including five important components, (1) discussing with patients understanding of cancer, (2) identifying objective and creating an ordering of important goals, (3) clarifying realistic short- and long-term goals achievable within the context of cancer, (4) recognizing the multiple pathways toward goals and selecting pathways with the highest chance of success, and (5) finding ways to increase agency and monitor their pathway to the goal [13]. In order to improve resilience, though stress management and resilience training, which is a brief, group-based cognitive behavioral therapy, patients were taught to redirect their perceptions of cancer and focus on adjustment and growth [14]. Interventions that targeted other aspects of the cancer experience (e.g., self-esteem, optimism, and self-efficacy) might in fact indirectly enhance resilience as well [46].

## Limitation

First, we used a convenient sample, which limited the generalizability of the findings to other cancer patients. Second, depression measured by the self-report of CES-D mainly referred to the depressive symptom in our study, and in-depth clinical evaluation should be employed to identify the depression. Third, data was mainly obtained using self-administered questions, so there was a possibility of recall and reporting bias. Forth, about the fact that we surveyed patients during an inpatient hospitalization, we cannot distinguish the special stressor in itself. Fifth, our study was cross-sectional, and thus we are unable to assess the causal relations among study variables. Additionally, depressive symptoms early on in prostate cancer diagnosis and treatment might be of clinical significance. Further longitudinal studies are needed to validate the current findings.

## Conclusions

PCa patients in China suffer from depressive symptoms (65.9%). Hope and resilience mediated the association between perceived social support and depressive symptoms. Perceived social support (especially support from family) and hope/resilience should be contained in depression preventions and treatments targeting PCa patients.

## Data Availability

The data were used under license for the current study, and so are not publicly available. Data are however available from the authors upon reasonable request and with the permission of the Shengjing Hospital.

## Abbreviations

CES-D: Center for Epidemiologic Studies Depression Scale
MSPSS: Multidimensional Scale of Perceived Social Support
AHS: Adult Hope Scale
